# Dizziness, Scanning Speech, and Transient Hyperreflexia as Atypical Initial Manifestations of Antiganglioside Q1b‐Negative Miller Fisher Syndrome: A Case Report and Literature Review

**DOI:** 10.1002/iid3.70580

**Published:** 2026-11-05

**Authors:** Wenjing Wu, Mengying Mao, Bin Xu, Liang'e Xu

**Affiliations:** ^1^ The Second Clinical Medical College Zhejiang Chinese Medical University Hangzhou Zhejiang P.R. China; ^2^ Departments of Neurology The Second Affiliated Hospital of Zhejiang Chinese Medical University Hangzhou Zhejiang P.R. China; ^3^ Departments of Neurology The Third Affiliated Hospital of Wenzhou Medical University Wenzhou Zhejiang P.R. China

**Keywords:** anti‐GQ1b‐negative, central nervous system, dizziness, hyperreflexia, Miller‐Fisher syndrome, scanning speech

## Abstract

**Background:**

Miller‐Fisher syndrome (MFS) is characterized by the triad of ataxia, areflexia, and ophthalmoplegia. To our knowledge, the combination of initial dizziness, scanning speech, transient hyperreflexia with a Babinski sign, and persistent anti‐GQ1b seronegativity has not been previously reported.

**Case Presentation:**

A 27‐year‐old Chinese Han male patient presented with prominent dizziness and scanning speech as the primary symptoms. Physical examination revealed hyperreflexia of all tendon reflexes initially, along with intention tremor and a positive Babinski's sign on the right side. Subsequently, he exhibited the classic triad of MFS, and cerebrospinal fluid analysis showed albuminocytological dissociation. He was ultimately diagnosed with MFS. Electromyography (EMG) revealed significantly abnormal blink reflexes, dysfunction of the brainstem auditory pathway, particularly within the central nervous system, and a descending sensory nerve action potential (SNAP) in the left sural nerve. Cerebral Magnetic Resonance Imaging (MRI) and ganglioside Q1b (GQ1b) antibodies were both negative. After treatment with intravenous immunoglobulin (IVIG) for 5 days, the patient's condition significantly improved. By the 1‐month follow‐up, his neurological function had completely recovered.

**Conclusion:**

The involvement of dizziness, scanning speech, hyperreflexia, and Babinski's sign in anti‐GQ1b‐negative MFS is quite rare, indicating early central nervous system (CNS) involvement. The pathogenesis of MFS may have a broader immunological basis rather than being solely attributable to a single autoantibody‐mediated response against gangliosides.

## Graphical abstract

This first report of Miller‐Fisher syndrome with dizziness and scanning speech as initial symptoms also showed CNS signs including hyperreflexia, intention tremor, and Babinski sign. Despite the eventual development of classic features and a favorable treatment response, the patient tested negative for anti‐GQ1b antibodies, suggesting a broader immunological mechanism.

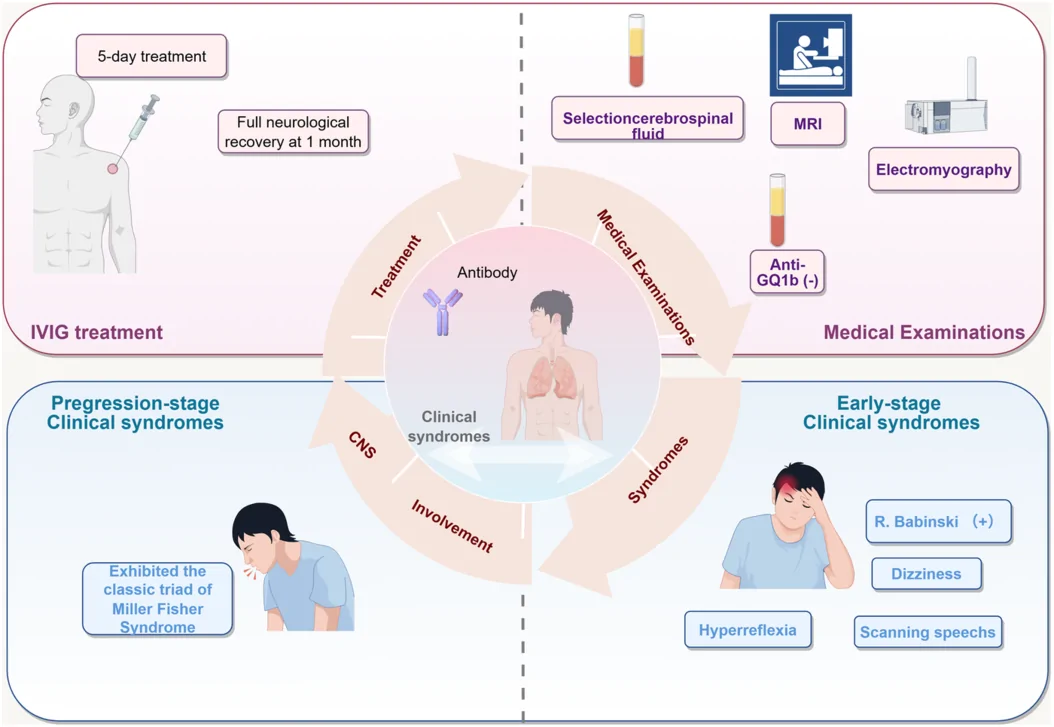

**Abbreviations**
AMAN: acute motor axonal neuropathy
BBE: Bickerstaff brainstem encephalitis
CNS: central nervous system
CSF: cerebrospinal fluid
EEG: Electroencephalogram
EMG: electromyogram
GAD: glutamic acid decarboxylase
GBS: Guillain‐Barre Syndrome
GQ1b: ganglioside Q1b
GSC: ganglioside complexes
IVIG: intravenous immunoglobulin
MFS: Miller‐Fisher syndrome
MRI: Magnetic Resonance Imaging
NMJ: neuromuscular junction
PET: positron emission tomography
PNS: peripheral nervous system
SEP: sensory evoked potential
SFEMG: Single‐fiber electromyography
SNAP: sensory nerve action potential
TMS: transcranial magnetic stimulation

## 1 Introduction

Miller Fisher Syndrome (MFS) was first described by Charles Miller Fisher in 1956 as a clinical triad of ataxia, areflexia, and ophthalmoplegia [1]. While traditionally considered a peripheral neuropathy, MFS and Bickerstaff brainstem encephalitis (BBE) are now recognized as overlapping components of the anti‐GQ1b antibody syndrome spectrum [2, 3]. These entities differ primarily by the predominance of central (CNS) versus peripheral (PNS) nervous system involvement. BBE is characterized by altered consciousness and/or pyramidal signs, whereas MFS typically presents with areflexia [4]. Consequently, the combination of dizziness and scanning speech, suggestive of brainstem and/or cerebellar involvement, represents a rare initial presentation in MFS. Herein, we describe a case in which dizziness, scanning speech, intention tremor, hyperreflexia, and a unilateral Babinski sign were the initial clinical manifestations of an anti‐GQ1b‐negative MFS.

## 2 Case Presentation

A 27‐year‐old male Chinese Han patient acutely developed persistent dizziness and dysarthria, accompanied by nausea and vomiting on Day 1. The symptoms progressed, and he experienced significant imbalance by Day 2. The patient was admitted to our hospital on Day 3. Upon admission, his vital signs were within normal limits. He reported ongoing dizziness and exhibited scanning speech, an unsteady gait, and diplopia on horizontal gaze. There were no indications of limb weakness or abnormal sensory perception. The patient denied any prior infections or vaccination history. His personal, family, reproductive, and past medical histories were noncontributory.

On physical examination, the patient was fully conscious and exhibited no signs of meningeal irritation. A limitation in bilateral abductive eye movement was observed, with no nystagmus present during horizontal or upward gaze. Both direct and indirect pupillary light reflexes were normal, and no eyelid ptosis was noted. The patient demonstrated noticeable dysarthria, characterized by scanning speech rather than slurred speech, along with an apparent intention tremor. Examination of other cranial nerves yielded no positive findings. No weakness or sensory deficit, including hypalgesia or impaired joint position or vibration sense, was detected in any of the limbs, and all tendon reflexes were hyperreflexive (3+). The patient displayed an ataxic gait, significant dysmetria, and failed the finger‐to‐nose and heel‐knee‐shin tests. Both the Romberg sign and right‐sided Babinski's sign were positive.

The aforementioned symptoms exhibited further progression on day 6. The patient developed bilateral eyelid ptosis and experienced restricted ocular movements, specifically in abduction and vertical gaze. Additionally, the patient's dizziness and scanning speech persisted, and the ataxic condition advanced to the point where the patient was unable to maintain a stable standing position. Significantly, all tendon reflexes were reduced to 0+. Throughout the course of his condition, he exhibited no disturbances in consciousness, and his clinical manifestations ultimately conformed to the classic Miller Fisher Syndrome (MFS) triad.

Serum white blood cell count was 9.69 × 10^9/L, with a neutrophil percentage of 72.4%, and CRP was 1.3 mg/L. Other laboratory investigations were within reference ranges. Upon inspecting the cerebrospinal fluid (CSF) analysis, we observed albuminocytological dissociation. We conducted the CSF and serum antibody analysis on Day 3 after onset. Antibodies against gangliosides (anti‐GD1a, anti‐GD1b, anti‐GM1, anti‐GM2, anti‐GM3, anti‐GT1b, and anti‐GQ1b), antibodies against neuromyelitis optica, oligoclonal bands, and paraneoplastic neurological syndrome‐associated antibodies were all negative. To ensure this was a truly anti‐GQ1b‐negative case, we detected GQ1b antibodies in the serum on Day 7 after the onset of symptoms and before treatment with IVIG; the results turned out to be negative. Routine and contrast‐enhanced MRI did not reveal any abnormal foci, including the brainstem, cerebellum, eyeballs, extraocular muscles, and orbits (Chart 1). The electroencephalogram (EEG) showed no epileptiform discharges. The EMG indicated significant abnormalities in blink reflexes and dysfunction of the brainstem auditory pathway (especially in the CNS), while the left sural nerve SNAP was descending. The patient was diagnosed with MFS based on the characteristic clinical triad after excluding other differential diagnoses. The patient received IVIG at a dose of 0.4 g/kg/day for 5 days. Clinical improvement was observed within 5 days of treatment initiation, with complete neurological recovery at the 1‐month follow‐up. Given the well‐recognized potential for spontaneous recovery in MFS, a causal relationship between IVIG and the favorable outcome cannot be inferred from this single case; controlled studies are required to establish treatment efficacy.

**Chart 1:**
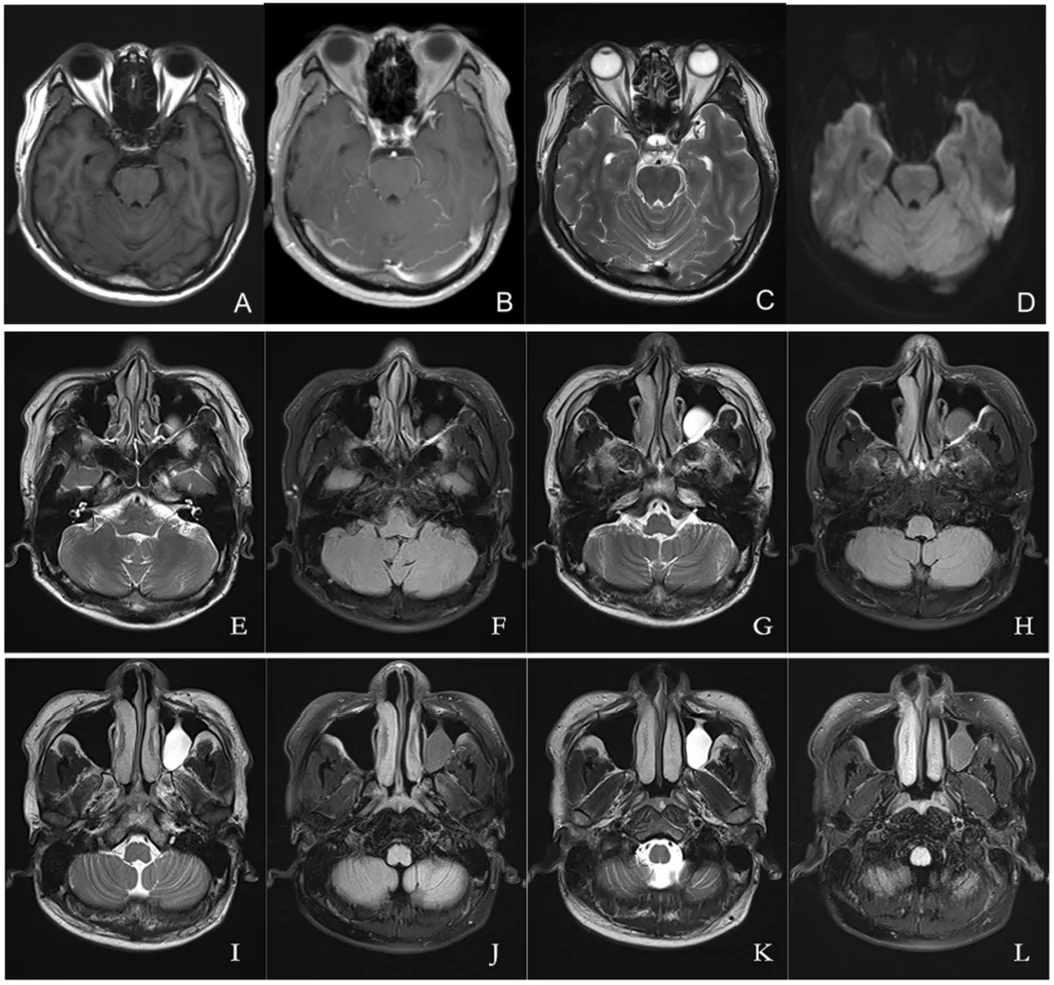
T1 WI axial scan (A), T1 WI contrast‐enhanced axial scan (B), T2 WI axial scan (C) and DWI axial scan (D) of brain MRI didn't show any abnormal focus including pons and cerebellum, and eyeball, extraocular muscle, orbit were normal. MRI‐T2 and FLAIR images (E–L) of the medulla oblongata were normal too.

## 3 Discussion

MFS, a variant of GBS, is defined by the classic clinical triad of ophthalmoplegia, ataxia, and areflexia. While these symptoms typically follow a bacterial or viral infection, our patient had no such history. Distinct from the ascending paralysis of GBS, MFS typically exhibits a descending progression starting with the cranial nerves [2]. Our patient initially presented with dizziness, scanning speech, ophthalmoplegia, and atypical hyperreflexia, with the full clinical triad emerging only later. Previous literature highlights the heterogeneity of MFS presentations; reported variations include dysarthria, ataxia, and dysphagia [5], reversible lingual weakness [6], and initial dizziness combined with unilateral visual loss [7] or ptosis [8]. Although dizziness and vestibular symptoms have been documented in MFS [9, 10], and dysarthria or bulbar palsy as atypical presentations have also been reported [5, 11], the specific initial constellation of prominent dizziness combined with scanning speech, transient hyperreflexia, intention tremor, and a unilateral Babinski sign in an anti‐GQ1b–negative patient remains distinctly unusual. Consequently, the distinctive features of this case lie not in the individual symptoms of dizziness or dysarthria, but in their specific combination with transient pyramidal signs and negative anti‐GQ1b serology.

The clinical presentation of MFS is frequently complicated by partial or variant forms, the underlying mechanisms of which remain poorly understood. Some cases overlap with GBS, such as acute pharyngeal weakness and pharyngeal‐cervical‐brachial (PCB) syndrome. While these localized GBS variants may share clinical features with MFS, they typically lack ataxia. Our patient exhibited no oropharyngeal, neck, or upper limb involvement, but eventually developed the classic triad of ophthalmoplegia, ataxia, and areflexia. Although the diagnosis of MFS was established according to the 2014 classification criteria [12], the transient pyramidal signs observed in our patient warrant further consideration regarding the distinction between MFS, Bickerstaff brainstem encephalitis (BBE), and MFS–BBE overlap. Current diagnostic frameworks recognize MFS and BBE as components of the anti‐GQ1b antibody syndrome spectrum, differing primarily by the degree of central versus peripheral nervous system involvement [13, 14]. BBE is characterized by altered consciousness and/or pyramidal signs, whereas MFS typically presents with areflexia; however, the distinction is not always clear‐cut, and overlap syndromes have been well documented [4, 13]. Our patient exhibited transient hyperreflexia and a unilateral Babinski sign on Day 3, which subsequently evolved into areflexia by Day 6, at which point the classic MFS triad was fully established; consciousness remained clear throughout the entire clinical course. This temporal evolution—initial pyramidal signs resolving as the classic MFS triad emerged—argues against pure BBE, which typically requires sustained altered consciousness or persistent pyramidal signs, and favors MFS with transient early central nervous system involvement rather than a formal MFS–BBE overlap.

Although traditionally viewed as a peripheral polyneuropathy, increasing evidence highlights CNS involvement in MFS. Kim et al. [15] have demonstrated cerebellar (vermis and hemispheres) glucose hypermetabolism in 10 patients with MFS, and the hypermetabolism was normalized on follow‐up PET with an improvement in ophthalmoplegia and ataxia in one patient. Similarly, cerebellar hypoperfusion on SPECT imaging [16] has been reported in MFS, providing additional radiological evidence for CNS involvement. Sandler et al. [17] demonstrated a reduced NAA/Cr ratio in the cerebellar vermis and right cerebral hemisphere by MRS, suggestive of cerebellar dysfunction, which normalized 2.5 months later in parallel with clinical recovery. Electrophysiological studies provide complementary evidence for brainstem involvement. Kuwabara et al. [18] systematically reviewed electrophysiological findings in Fisher syndrome and demonstrated that blink‐reflex and brainstem auditory evoked‐potential (BAEP) abnormalities frequently reflect central pathway dysfunction. Alberti et al. [19] reported early electrophysiological abnormalities in Fisher‐Bickerstaff syndrome, further supporting subclinical central involvement. The Neurophysiological findings of our patient also provided further evidence of brainstem involvement. The blink reflex demonstrated absent bilateral R2 responses, whereas the R1 responses were preserved with low amplitude and normal latency—a pattern indicating dysfunction of the central pontine and medullary reflex pathways rather than of the peripheral trigeminal or facial nerves. Similarly, BAEP revealed a prolonged right‐sided III–V interpeak latency with a normal I–III interval, localizing the dysfunction predominantly to the central brainstem auditory pathway, namely the upper pons and midbrain. Taken together with the transient hyperreflexia and unilateral Babinski sign observed on clinical examination, these neurophysiological findings suggest transient corticospinal tract and central brainstem involvement, supporting the presence of early CNS involvement in this patient.

Although previous studies have demonstrated a high prevalence of anti‐GQ1b IgG antibodies in MFS patients (83%–95%) [20], the absence of anti‐GQ1b antibodies should not preclude a diagnosis of MFS. A subset of MFS patients may test positive for antibodies against other single gangliosides or ganglioside complexes (GSC), such as GM1, Asialo‐GM1, GD1, GM1b, GD1c, or GalNAc‐GM1b. Furthermore, anti‐ganglioside antibodies remain negative in a minority of MFS cases. Ioannis E. Dagklis and colleagues reported a typical MFS case with relatively elevated anti‐glutamic acid decarboxylase (GAD) antibodies; follow‐up evaluations revealed continuous clinical improvement correlated with the decline and eventual normalization of anti‐GAD levels [21]. Similar cases of anti‐GAD‐positive MFS involving central nervous system (CNS) pathology have also been described in recent reports [22, 23]. In the present case, the patient exhibited early CNS involvement followed by progression to typical MFS, despite testing negative for both anti‐GQ1b and anti‐GSC antibodies. Therefore, contrary to the proposed “anti‐GQ1b antibody syndrome” framework, we hypothesize that the pathogenesis of this clinical spectrum may involve a broader immunological basis rather than being limited to a single autoantibody‐mediated response against gangliosides. Nevertheless, this hypothesis remains speculative; definitive conclusions regarding alternative immunological mechanisms in anti‐GQ1b‐negative MFS will require multicenter prospective studies incorporating expanded autoantibody panels and high‐throughput immunoproteomic approaches.

## 4 Conclusion

In conclusion, MFS may initially present with prominent dizziness, scanning speech, hyperreflexia, and intention tremor, features suggesting concurrent peripheral and central involvement; these atypical manifestations should prompt early consideration of MFS even when anti‐GQ1b antibodies are undetectable, as seronegativity in serum or CSF does not exclude the diagnosis. Our case further supports the notion that MFS may rest on a broader immunological basis than injury mediated by a single anti‐ganglioside antibody. Future multicenter prospective cohorts, integrating expanded autoantibody and immunoproteomic profiling with longitudinal multimodal neurophysiological and neuroimaging assessments, are warranted to delineate the full spectrum of anti‐GQ1b‐negative MFS and MFS–BBE overlap, to clarify the mechanisms of CNS involvement, and to evaluate the efficacy of IVIG together with biomarkers predictive of disease course and treatment response.

## Author Contributions

**Wenjing Wu:** data curation, writing – original draft, investigation. **Mengying Mao:** writing – review and editing, data curation. **Bin Xu:** writing – review and editing, investigation. **Liang'e Xu:** data curation, writing – original draft, writing – review and editing, formal analysis.

## Funding

The authors have nothing to report.

## Ethics Statement

The study was conducted in accordance with the Declaration of Helsinki and approved by the Ethics Committee of Ruian People's Hospital (Approval No. YJ2026140).

## Consent

Written informed consent was obtained from the patient for publication of this case report. A copy of the written consent is available for review by the Editor of this journal.

## Conflicts of Interest

The authors declare no conflicts of interest.

## Supporting information


Supporting File

## Data Availability

The data that support the findings of this study are available from the corresponding author upon reasonable request. The data sets used or analyzed during this study are available from the corresponding author on reasonable request.
