# Supplementary material for: Dizziness, Scanning Speech, and Transient Hyperreflexia as Atypical Initial Manifestations of Antiganglioside Q1b‐Negative Miller Fisher Syndrome: A Case Report and Literature Review
Source: Immun Inflamm Dis. 2026 Nov 5;14(11):e70580. doi: 10.1002/iid3.70580 (PMC13737464; doi:10.1002/iid3.70580)
Supplement: Supplementary file 1 [file IID3-14-e70580-s001.docx]

同意发表声明

由于您所患疾病 Miller-Fisher 综合征的临床表现比较不典型 ，我们将发表一篇关于您的诊治过程的报道并展开讨论分析，以便对您所患疾病的能有更深入的了解和更丰富的经验总结。该报道将公开描述您的性别、年龄和发病的具体临床症状、体征变化情况以及一些相关的实验室检验和辅助检查结果，但不会公开您的姓名、联系方式等个人私密信息。同时，该研究不用于商业性活动，不会改变您的临床诊疗方案，也不造成额外的治疗费用及创伤。您本人及家属是否同意将相关临床数据用于发表，请选择并签字如下：

同意☑ 不同意☐

签名：黄彬彬

日期：2025.1.12

Statement of Consent for Publication

The manifestations of Miller-Fisher syndrome in your case are atypical; th
us, we have decided to publish a report discussing the process of your diagnosi
s and treatment to enhance the understanding of this disease. The report will in
clude details regarding your gender, age, and the onset of specific clinical sym
ptoms and signs, as well as relevant laboratory tests and special examination re
sults. However, your personal information, such as your name or contact infor
mation, will not be published. Furthermore, the clinical data will not be used fo
r commercial purposes. This will not affect your clinical treatment, nor will it i
ncur any additional treatment costs or bodily harm. If you agree to the use of th
e relevant clinical data for publication, please sign your name below:

Yes☑ No☐

Sign name: Binbin Huang

Date: 2025.1.12
